# Exploring Systemic Autoimmunity in Thyroid Disease Subjects

**DOI:** 10.1155/2018/6895146

**Published:** 2018-12-17

**Authors:** Thushani Siriwardhane, Karthik Krishna, Vinodh Ranganathan, Vasanth Jayaraman, Tianhao Wang, Kang Bei, John J. Rajasekaran, Hari Krishnamurthy

**Affiliations:** ^1^Vibrant America LLC, San Carlos, CA, USA; ^2^Vibrant Sciences LLC, San Carlos, CA, USA

## Abstract

**Introduction:**

Individuals with one autoimmune disease are at risk of developing a second autoimmune disease, but the pathogenesis or the sequential occurrence of multiple autoimmune diseases has not been established yet. In this study, we explored the association and sequential occurrence of antibodies in thyroid disease and systemic autoimmune disease subjects. We evaluated thyroid hormones, thyroid-stimulating hormone (TSH), free thyroxine (FT4), thyroid autoantibodies, anti-thyroperoxidase (anti-TPO), and anti-thyroglobulin (Tg) to comprehend the association with systemic autoimmune autoantibodies, anti-nuclear antibodies (ANA), and autoantibodies to extractable nuclear antigens (ENA) in subjects with thyroid-related symptoms.

**Methods:**

A total of 14825 subjects with thyroid-related symptoms were tested at Vibrant America Clinical Laboratory for thyroid markers (TSH, FT4, anti-TPO, and anti-Tg) and an autoimmune panel (ANA panel and ENA-11 profile) from March 2016 to May 2018. Thyroid-positive (based on TSH and FT4 levels), anti-TPO-positive, and anti-Tg-positive subjects were assessed for the prevalence of ANA and anti-ENA antibodies. A 2-year follow-up study was conducted to assess the sequential order of appearance of autoimmune markers in thyroid and systemic autoimmune diseases.

**Results:**

In the retrospective analysis, 343/1671 (20.5%), 2037/11235 (18.1%), and 1658/9349 (17.7%) of thyroid+, anti-TPO+, and anti-Tg+ subjects were found to be seropositive for ANA. Anti-ENA was detected in a higher prevalence than ANA with 475/1671 (28.4%), 3063/11235 (27.3%), and 2511/9349 (26.9%) in the same groups of subjects, respectively. Our results are found to be much higher than the reported prevalence of anti-ENA in general population. During the 2-year follow-up study, anti-TPO appeared significantly earlier than ANA and anti-ENA in an average of 253 (±139) and 227 (±127) days, respectively.

**Conclusions:**

A high prevalence of anti-ENA and ANA was found to be coexisting with autoimmune thyroid disease subjects, with anti-TPO occurring prior to the onset of ANA and anti-ENA. Therefore, frequent follow-ups and evaluation of ANA and anti-ENA in subjects with anti-TPO positivity would be beneficial in early detection of other systemic autoimmune diseases.

## 1. Introduction

Autoimmune diseases are common in Western countries with a prevalence ranging from 7.6 to 9.4% [1–3], affecting individuals' chronic morbidity, quality of life, and health care costs. It occurs more frequently in women and is one of the leading causes of death among young to middle-aged women [4]. Autoimmune diseases are mostly controlled by environmental triggers in genetically susceptible individuals [5]. Unfortunately, one disorder of autoimmune pathogenesis can lead to additional autoimmune diseases. About 25% of patients with one autoimmune disease have the tendency for the coexistence of other autoimmune diseases [6].

Autoimmune thyroid disease (AITD) is one of the common representatives in the autoimmune disease spectrum [7]. AITD is usually recognized by the presence of anti-thyroid peroxidase (TPO) and anti-thyroglobulin (Tg) autoantibodies in conjunction with thyroid hormone disparity [8]. Hashimoto's disease and Grave's disease are the most common causes for hypothyroidism and hyperthyroidism in AITD, respectively [7]. The prevalence of AITD in other autoimmune disease patients such as celiac disease, Sjogren's syndrome, systemic lupus erythematosus (SLE), and rheumatoid arthritis has been studied widely [9, 10]. Antinuclear antibodies (ANA) and the antibodies against extractable nuclear antigens (ENA) are recognized as valuable diagnostic markers in evaluating systemic autoimmune diseases (e.g., systemic lupus erythematosus and mixed connective tissue disorder). However, information on the sequence of the occurrence of autoantibodies in patients with these multiple autoimmune diseases is relatively lacking.

Longitudinal assessment of autoantibodies against AITD and systemic autoimmune disease will allow us to understand the sequential appearance of these autoantibodies. The presence of one autoimmune disease marker will be a predictive factor for the other autoimmune disorder and hence will be useful for close monitoring, arranging frequent follow-up testing, and improving therapeutic measurements. In this study, we sought to identify the sequential appearance of AITD autoantibodies and systemic autoimmune antibodies to understand the correlation of these markers in subjects with multiple autoimmune disease conditions. Understanding the sequential occurrence of autoantibodies will provide autoimmune disease prediction in subjects with multiple autoimmune symptoms.

## 2. Material and Methods

### 2.1. Patient Selection and Study Design

A total of 14825 subjects with thyroid-related symptoms were tested at Vibrant America Clinical Laboratory for thyroid markers (TSH, FT4, anti-TPO, anti-Tg) and by an ANA panel and ENA-11 profile from March 2016 to May 2018. This retrospective analysis was completed using deidentified laboratory test results.

For analysis, subjects were subcategorized into the following groups:

1. Thyroid-positive subjects (thyroid+)—subjects who were hypothyroid or hyperthyroid based on their TSH and FT4 levels (either subclinical or overt)
2. Anti-TPO-positive subjects (anti-TPO+)—subjects who were seropositive for anti-TPO
3. Anti-Tg-positive subjects (anti-Tg+)—subjects who were seropositive for anti-Tg

### 2.2. TSH, FT4, Anti-TPO, and Anti-Tg Tests

TSH, FT4, anti-TPO, and anti-Tg were measured using the commercial Roche e601 analyzer (Roche Diagnostics, Indianapolis, IN, USA) according to the manufacturer's recommendations. All reagents were purchased from Roche Diagnostics (Indianapolis, IN, USA). Human serum specimens were used on Elecsys immunoassay analyzers.

Specific TSH monoclonal antibodies specifically directed against human TSH were employed in the Elecsys TSH assay. The antibodies labeled with a ruthenium complex consist of a chimeric construct from human- and mouse-specific components. As a result, interfering effects due to HAMA (human anti-mouse antibodies) were largely eliminated.

The Elecsys FT4 test employed a specific anti-T4 antibody labeled with a ruthenium complex to determine the free thyroxine. The quantity of antibody used was so small (equivalent to approx. 1-2% of the total T4 content of a normal serum sample) that the equilibrium between bound and unbound T4 remained virtually unaffected.

Elecsys anti-TPO assay employed recombinant antigens and polyclonal anti-TPO antibodies whereas Elecsys anti-Tg assay employed monoclonal human anti-Tg antibodies.

The internal quality control procedures for each test can be found in the supplementary material (available here).

### 2.3. Reference Ranges for Thyroid Markers

Thyroid hormone reference ranges are subject to the lab where the test is performed. In this study, we used the reference ranges that majority of the commercial test labs and hospital labs use. The reference ranges of thyroid markers in a healthy control used in this study are shown in Table 1.

The categorization of serologic thyroid positivity by evaluating TSH and FT4 levels used in this study is shown in Table 2.

### 2.4. ANA Panel

ANA detection was performed with Vibrant™ ANA HEp-2 (Vibrant America LLC, San Carlos, CA, USA), which is a solid-phase biochip immunofluorescence assay designed to detect antinuclear antibodies. The results were interpreted based on the ANA pattern observed, the titer of the autoantibody, and the age of the patient. A sample was considered ANA negative (ANA−) if specific staining was equal to or less than a negative control (buffer containing preservative and human serum with no IgG antinuclear antibodies). A sample was considered ANA positive (ANA+) if any specific staining (homogeneous, centromere, speckled, nucleolar, and peripheral) was observed to be greater than that of the negative control. A 1 : 40 dilution was suggested as a good dilution for ANA screening with the visibility of a pattern; however, low-titer positive results might occur in apparently healthy persons. Therefore, the patient's total clinical profile should always be considered when interpreting ANA results. The internal quality control procedure for the ANA panel can be found in the supplementary material.

### 2.5. ENA-11 Profile

The ENA-11 profile included testing antibodies for SSA(Ro), SSB(La), RNP/Sm, Jo-1, Sm, Scl-70, chromatin, centromere, histone, RNA polymerase III, and dsDNA. A solid-phase biochip immunofluorescence assay was used to detect antibodies for SSA(Ro), SSB(La), RNP/Sm, and Jo-1 that reports qualitative and semiquantitative results of IgG to SSA(Ro), SSB(La), RNP/Sm, Jo-1. Patient results were interpreted by comparison with calibrators, controls, and cutoff values. The results were interpreted according to the international guidelines announced by the European Autoimmunity Standardization Initiative and the International Union of Immunologic Societies/World Health Organization/Arthritis Foundation/Centers for Disease Control and Prevention autoantibody standardizing committee [11].

The results for Sm, Scl-70, chromatin, centromere, histone, and RNA polymerase III were measured using a commercially available ELISA kit (Inova Diagnostics Inc., San Diego, CA, USA) according to the manufacturer's recommendations. The internal quality control procedure for the ENA panel can be found in the supplementary material.

### 2.6. Statistical Analysis

The retrospective study was performed using clinical data from the deidentified subjects and analyzed using Java for Windows version 1.8.161. Data were expressed as mean ± standard deviation (SD). Multiple logistic regression analysis was performed to evaluate the association between the autoimmune markers. *P* < 0.05 was considered significant.

## 3. Results

### 3.1. Prevalence of ENA and ANA in Thyroid Marker-Positive Subjects

**Figure 1:**
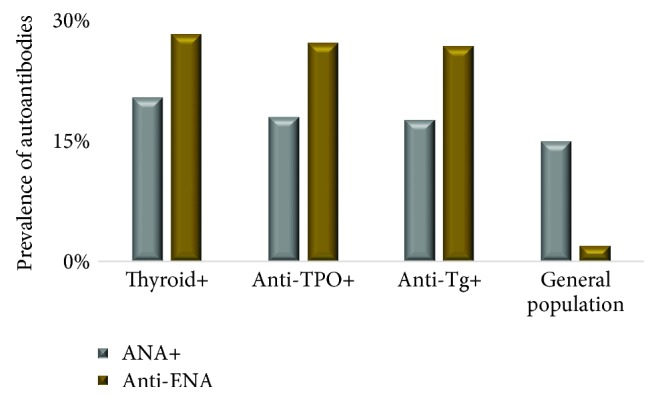
Prevalence of ANA and anti-ENA autoantibodies in thyroid (ANA: 343/1671, anti-ENA: 475/1671), anti-TPO (ANA: 2037/11235, anti-ENA: 3063/11265), and ant-Tg (ANA: 1658/9349, anti-ENA: 2511/9349) positive subjects.

As shown in Table 3 and Figure 1, the prevalence of ANA and anti-ENA was assessed in thyroid+, anti-TPO+, and anti-Tg+ subjects.

The prevalence of ANA was similar in subjects who were thyroid positive (20.5%), anti-TPO positive (18.1%), and anti-Tg positive (17.7%). Of the 343 subjects who were serologically thyroid positive, 221 (64.4%) were hypothyroid subjects and 122 (35.6%) were hyperthyroid subjects. The prevalence of anti-ENA in thyroid-positive subjects was 28.4%, and of them 321 (67.6%) were hypothyroid subjects, and 154 (32.4%) were hyperthyroid subjects. Also, anti-ENA was measured in subjects who were positive for anti-TPO and anti-Tg with a prevalence of 27.3% and 26.9%, respectively.

**Figure 2:**
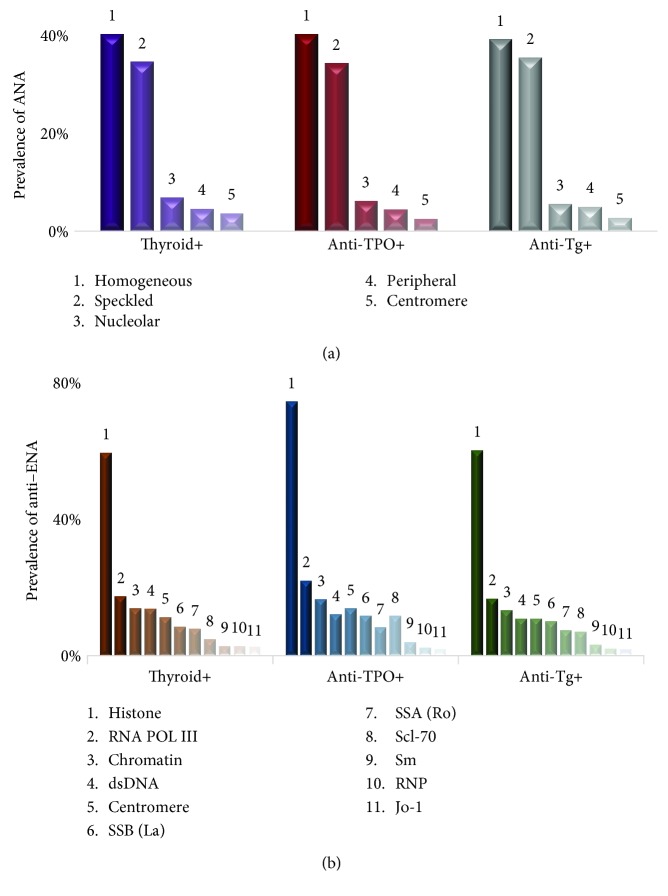
Prevalence of subcategories of (a) ANA and (b) anti-ENA in thyroid (*n* = 343), anti-TPO (*n* = 2037), and anti-Tg (*n* = 1658) positive subjects.

Next, we assessed the subcategories of ANA and anti-ENA autoantibodies found in thyroid-, anti-TPO-, and anti-Tg-positive subjects. Five ANA patterns were seen among the subjects evaluated in this study. As shown in Figure 2(a), all three groups had “homogenous” ANA pattern as the most frequent pattern followed by speckled, nucleolar, peripheral, and centromere. A detailed analysis of each ANA pattern in each group was presented in Table 4.

All 11 anti-ENA markers were present in thyroid+, anti-TPO+, and anti-Tg+ subjects. As shown in Figure 2(b), anti-histone was the most frequently found anti-ENA marker in all three groups with a frequency of 57.9%, 72.4%, and 58.6% in thyroid+, anti-TPO+, and anti-Tg+ subjects, respectively. Sm, RNP, and Jo-1 are the least prevalent in all three groups, but other marker's position of prevalence varied in each group. A detailed analysis of each marker present in each group was tabulated in Table 5.

### 3.2. Early Detection of Anti-TPO Predicts ENA and ANA Conversion

**Figure 3:**
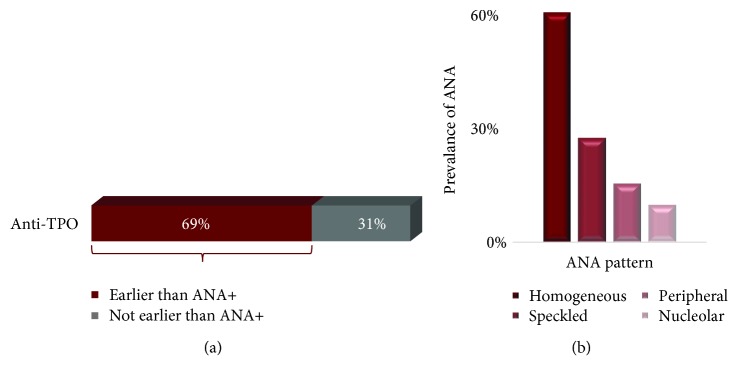
(a) Prevalence of anti-TPO occurrence prior to the onset of ANA. Anti-TPO was positive in 51/74 subjects 253 (±139) days prior to the onset of ANA positivity. (b) The distribution of subcategories of ANA that has anti-TPO earlier than ANA.

We selected a cohort of 74 subjects who had negative test indexes on their first visit for ANA profile but showed positive ones in their following visits. The thyroid panel was tested on these subjects to identify any thyroid marker that precedes the anti-ENA positivity. TSH, FT4, or anti-Tg were not significantly expressed ahead of ANA positivity but as shown in Figure 3, anti-TPO positivity was preceding ANA positivity in 51 subjects (69%) in an average time of 253 (±139) days. The homogeneous pattern was the most frequent pattern found in cohort with a most frequent titer of 1 : 40.

**Figure 4:**
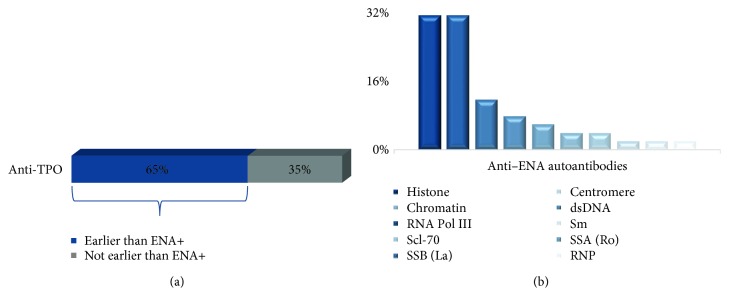
(a) Prevalence of anti-TPO occurrence prior to the onset of anti-ENA. Anti-TPO was positive in 51/78 subjects 227 (±127) days prior to the onset of anti-ENA positivity. (b) The distribution of subcategories of anti-ENA that has anti-TPO earlier than anti-ENA.

Next, we evaluated a cohort of 78 subjects whose initial ENA test index was negative but had turned positive in subsequent follow-up visits. The thyroid panel was tested on these subjects to identify any thyroid marker that precedes the anti-ENA positivity. TSH, FT4 hormones, and anti-Tg autoantibody did not appear to be present prior to the onset of anti-ENA autoantibodies. But, as shown in Figure 4, anti-TPO showed positivity in 51 subjects (65%) for an average of 227 (±127) days ahead of anti-ENA positivity. The highest frequent anti-ENA marker was histone and RNA Pol III (31.4% each) followed by SSB(La) (11.8%). Jo-1 was not expressed in the subjects in this cohort. The least frequent markers were RNP (2%) and Sm (2%).

Finally, to confirm that anti-TPO appears ahead of ANA and anti-ENA and not vice versa, we measured ANA and anti-ENA profiles in subjects who had negative test indexes for anti-TPO at their first visit but were converted to positive ones in their subsequent visits. The data did not show any significant relationship with either ANA or anti-ENA occurring ahead of anti-TPO (data not shown).

## 4. Discussion

Autoimmune diseases are complex disorders caused by a combination of genetic susceptibility and environmental factors that may disrupt the immune system by attacking self-organs. These disruptions can create the path for future development of autoimmune diseases making patients with one autoimmune disease vulnerable for other autoimmune diseases [6, 12].

In our retrospective analysis, we evaluated the association between subjects with AITD markers and systemic autoimmune disease markers. Thyroid-positive subjects were categorized by assessing their TSH and FT4 levels despite the presence/absence of thyroid autoantibodies. Anti-TPO- and anti-Tg-positive subjects despite their thyroid hormone levels were evaluated separately since their association with AITD is directly related than thyroid hormones [13]. The prevalence of ANA and anti-ENA autoantibodies was assessed in subjects with thyroid positivity, anti-TPO positivity, and anti-Tg positivity. The prevalence of ANA which is the cornerstone marker in systemic autoimmune disease [14] was found to be 20.4% in thyroid-positive subjects, 18.0% in anti-TPO-positive subjects, and 17.6% in anti-Tg-positive subjects. Several other groups have conducted studies on the prevalence of AITD on systemic autoimmune patients [15, 16], but the prevalence of systemic autoantibodies in thyroid disease-related subjects is limited. In one study, Tektonidou et al. reported that the prevalence of ANA in AITD patients was as high as 35% [17], but in a separate study, Morita et al. reported it to be 26% [18] which is closer to our results for thyroid-positive subjects. But none of these studies were able to report a detailed prevalence on both ANA patterns and anti-ENA antibodies present in the same cohort of AITD subjects which could be beneficial in categorizing the specific systemic autoimmunity. We were able to provide a detailed analysis on the prevalence of ANA and anti-ENA in the same cohort of AITD subjects in a large population size of 14825 subjects. All three groups in our study with AITD markers (thyroid positive, anti-TPO positive, and anti-Tg positive) had homogenous as their most frequent ANA pattern. The homogenous pattern is more common in people with systemic lupus but also can be found in patients with mixed connective tissue disorder and drug-induced lupus.

The prevalence of anti-ENA autoantibodies was reported to be less than 2% in general population [19]; however, our study found it to be higher in thyroid-positive (28.3%), anti-TPO-positive (27.2%), and anti-Tg-positive subjects (26.8%). Also, the prevalence of anti-ENA was comparatively higher than the prevalence of ANA in thyroid positive, anti-TPO positive, and anti-Tg positive subjects. This result is consistent with the findings of Yang et al. which states that anti-ENA can be detected years earlier than ANA [20]; hence, there is a high possibility that the ANA disparity would increase with time for this cohort. The most prevalent anti-ENA subcategory was histone antibody with a frequency of 57.9% in thyroid-positive, 72.5% in anti-TPO-positive, and 58.6% in anti-Tg-positive subjects. These findings on high frequency of histone antibodies in subjects with positive AITD markers further support the recent research findings on the involvement of aberrant histone modifications in AITD pathogenesis [21].

The studies reported on the order of appearance of AITD and systemic autoimmune diseases are debatable [9, 22, 23]. The order of appearance of AITD and systemic autoimmune diseases had been reported in one study, but with a very low population size (*n* = 4) [24]. We have reported here the most comprehensive analysis with 14825 subjects to show the order of emergence of the systemic autoimmunity in AITD subjects. Our study shows that anti-TPO autoantibodies could be detected prior to the onset of systemic autoimmune disease antibodies, ANA and anti-ENA. Anti-TPO was present in 69% and 65% of subjects prior to the onset of ANA and anti-ENA antibodies, respectively. Anti-TPO was present 253 (±139) days prior to the onset of ANA and 227 (±127) days prior to the onset of anti-ENA. The average time of appearance of ANA is later than anti-ENA since anti-ENA may have the potential to be detected earlier than ANA [20]. The negative results in our control experiment on evaluating the presence of anti-ENA and ANA prior to the onset of anti-TPO or anti-Tg further confirmed that anti-TPO precedes the systemic autoimmune disease autoantibodies and not vice versa; thus, AITD may be a leading cause to the second systemic autoimmune disease. Moreover, histone antibody was again found to be one of the most frequent anti-ENA in converted subjects, further proving that histone modifications may play a role in AITD.

In conclusion, our data shows a strong association of the coexistence of AITD markers and systemic autoimmune markers and the presence of anti-TPO prior to the onset of ANA and anti-ENA. Therefore, routine evaluation of ANA and anti-ENA would be beneficial in subjects with positive anti-TPO for early detection of other systemic autoimmune diseases.

## Figures and Tables

**Table 1:** Reference ranges for thyroid markers studied.

Marker	Reference range
TSH	0.3–4.2 mIU/L
FT4	0.9–1.7 ng/dL
Anti-TPO	<9.0 IU/mL
Anti-Tg	<4.0 IU/mL

**Table 2:** Thyroid disease categorization.

Disease condition	TSH	FT4
Hypothyroidism		
Subclinical hypothyroidism	>4.2 mIU/L	0.9–1.7 ng/dL
Overt hypothyroidism	>4.2 mIU/L	<0.9 ng/dL
Hyperthyroidism		
Subclinical hyperthyroidism	<0.3 mIU/L	0.9–1.7 ng/dL
Overt hyperthyroidism	<0.3 mIU/L	>1.7 ng/dL

**Table 3:** Clinical characteristics of thyroid+, anti-TPO+, and anti-Tg-positive subjects.

	Thyroid+	Anti-TPO+	Anti-Tg+
Age (*X* ± SD)	50 ± 17	47 ± 16	47 ± 17
Sex	1268 F/403 M	7908 F/3327 M	6675 F/2674 M
ANA+	343/1671 (20.5%)	2037/11235 (18.1%)	1658/9349 (17.7%)
Anti-ENA+	475/1671 (28.4%)	3063/11265 (27.3%)	2511/9349 (26.9%)

**Table 4:** Frequency of ANA in each group.

ANA marker	Thyroid+ (*n* = 343)		Anti-TPO+ (*n* = 2037)		Anti-Tg+ (*n* = 1658)	
	Frequency	Percentage	Frequency	Percentage	Frequency	Percentage
Homogeneous	141	41.1%	835	50.0%	663	40.0%
Speckled	121	35.3%	711	35.0%	599	36.1%
Nucleolar	24	7.0%	129	6.3%	95	5.7%
Peripheral	16	4.7%	94	4.6%	85	5.1%
Centromere	13	3.8%	53	2.6%	46	2.8%

**Table 5:** Frequency of anti-ENA in each group.

Anti-ENA marker	Thyroid+ (*n* = 475)		Anti-TPO+ (*n* = 3063)		Anti-Tg+ (*n* = 2511)	
	Frequency	Percentage	Frequency	Percentage	Frequency	Percentage
Histone	275	57.9%	1798	72.5%	1471	58.6%
RNA POL III	80	16.8%	532	21.4%	408	16.2%
Chromatin	64	13.5%	398	16.0%	321	12.8%
dsDNA	63	13.3%	291	11.7%	265	10.6%
Centromere	52	10.9%	332	13.4%	265	10.6%
SSB (La)	39	8.2%	281	11.3%	244	9.7%
SSA (Ro)	36	7.6%	199	8.0%	182	7.2%
Scl-70	22	4.6%	215	11.3%	173	6.9%
Sm	13	2.7%	97	3.9%	77	3.1%
RNP	13	2.7%	59	2.4%	51	2.0%
Jo-1	12	2.5%	46	1.9%	44	1.8%

## Data Availability

The deidentified laboratory test data used to support the findings of this study are available from the corresponding author upon request.

## Supplementary Materials

Supplementary Materials Supplementary material contains descriptions of internal quality control procedures for TSH, free FT4, anti-TPO, anti-Tg, ANA panel, and ENA panel.
