# Supplementary material for: Exploring Systemic Autoimmunity in Thyroid Disease Subjects
Source: J Immunol Res. 2018 Dec 17;2018:6895146. doi: 10.1155/2018/6895146 (PMC6399525; doi:10.1155/2018/6895146)
Supplement: Supplementary Materials — Supplementary material contains descriptions of internal quality control procedures for TSH, free FT4, anti-TPO, anti-Tg, ANA panel, and ENA panel. [file 6895146.f1.pdf]

Exploring Systemic Autoimmunity in Thyroid Disease Subjects

Thushani Siriwardhane[1*], Karthik Krishna[2], Vinodh Ranganathan[2], Vasanth Jayaraman[2], Tianhao Wang[2], Kang Bei[2], John J. Rajasekaran[2], Hari Krishnamurthy[2*]

[1]*Vibrant America LLC., San Carlos, CA, USA*

[2]*Vibrant Sciences LLC., San Carlos, CA, USA*

Running title: Association of ANA and ENA in Thyroid-Related Disorders

Address correspondence to: Thushani Siriwardhane, PhD, Vibrant America LLC., 1360 Bayport Ave, San Carlos, CA 94070, USA. Phone: 650-508-8262, Fax: 650-508-8262. E-mail: thushanis@vibrant-america.com; Hari Krishnamurthy, Vibrant Sciences LLC., 1021 Howard Avenue, Suite B, San Carlos, CA 94070, USA. Phone: 1-866-364-0963, Fax: 1-650-508-8262. E-mail: hari@vibrantsci.com

DECLARATION OF CONFLICT OF INTEREST

Siriwardhane is an employee of Vibrant America LLC. Krishna, Ranganathan, Jayaraman, Wang, Bei, Rajasekaran, Krishnamurthy, are employees of Vibrant Sciences LLC.

Supplementary Material

Internal Quality Control

TSH

The test kits, Elecsys PreciControl TSH or PreciControl Universal (Roche Diagnostics Ltd, USA) was used for internal quality control measurements according to manufactures instructions. Controls for various concentrations were performed individually at least once every 24 hours when the test is in use, once per reagent kit, and following each calibration. Corrective measures were taken if values fall outside the defined limits.

Free FT4

The test kit, Bio-Rad Liquichek Immunoassay Plus control (Levels 1 and 3) (Bio-Rad, USA) was used for internal quality control measurements according to manufactures instructions. Controls for various concentrations were performed individually at least once every 24 hours when the test is in use, once per reagent kit, and following each calibration. Corrective measures were taken if values fall outside the defined limits.

Anti-TPO

The test kits, Elecsys PreciControl Thyro AB (Roche Diagnostics Ltd, USA) or Bio-Rad Immunoassay Specialty control (Bio-Rad, USA) was used for internal quality control measurements according to manufactures instructions. Controls for various concentrations were performed individually at least once every 24 hours when the test is in use, once per reagent kit, and following each calibration. Corrective measures were taken if values fall outside the defined limits.

Anti-Tg

The test kits, Elecsys PreciControl Thyro AB (Roche Diagnostics Ltd, USA) was used for internal quality control measurements according to manufactures instructions. Controls for various concentrations were performed individually at least once every 24 hours when the test is in use, once per reagent kit, and following each calibration. Corrective measures were taken if values fall outside the defined limits.

ANA Panel

A positive and a negative control was tested every time an assay was performed. A negative control was characterized by the absence of specific fluorescence background staining of all cells due to counterstain. A positive control (homogeneous pattern) was characterized by bright fluorescence. The homogeneous staining pattern is a diffused uniform staining of the entire nucleus. The controls were tested prior to evaluating the test sample. If controls fall outside the defined values, results were marked as invalid and the test was repeated.

ENA Panel

The calibrator was performed in triplicate each time the assay was tested. A negative control and a positive control were included in each assay. For each assay, the Index value of negative controls and the positive controls were confirmed to be within the laboratory established range. The test was repeated if the controls or calibrator failed.
